# Supplementary material for: Function-oriented design of robust metal cocatalyst for photocatalytic hydrogen evolution on metal/titania composites
Source: Nat Commun. 2021 Jan 8;12:158. doi: 10.1038/s41467-020-20464-x (PMC7794313; doi:10.1038/s41467-020-20464-x)
Supplement: Supplementary file 1 — Supplementary Information [file 41467_2020_20464_MOESM1_ESM.pdf]

Supplementary Information

Function-oriented design of robust metal cocatalyst for photocatalytic hydrogen evolution on metal/titania composites

Dong Wang[1,*] and Xue-Qing Gong[1]

[1]Key Laboratory for Advanced Materials and Joint International Research Laboratory for Precision Chemistry and Molecular Engineering, Feringa Nobel Prize Scientist Joint Research Center, Centre for Computational Chemistry and Research Institute of Industrial Catalysis, School of Chemistry and Molecular Engineering, East China University of Science and Technology, 130 Meilong Road, Shanghai, 200237, P. R. China.

*Corresponding author: wangd@ecust.edu.cn

The supplementary information includes:

Supplementary Fig. 1-8

Supplementary Table 1-2

1. Supplementary Figures

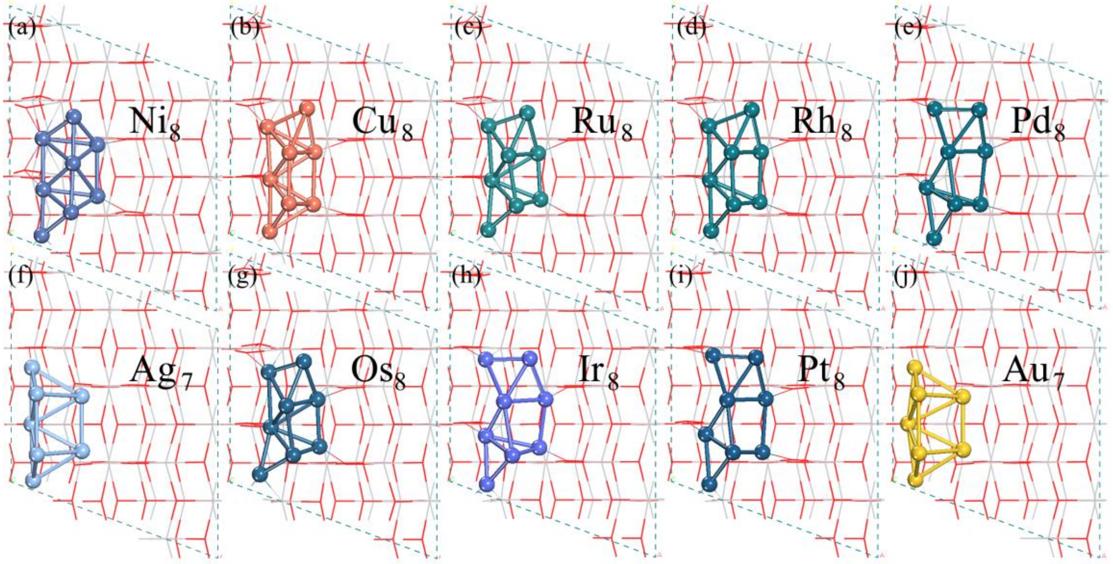

Supplementary Fig. 1. Optimized composite structures of candidate electron transfer metals (displayed by ball-and-stick) on TiO_2 support (line) using M_8/TiO_2 or M_7/TiO_2 models. Pannels (a-j) correspond to Ni_8/TiO_2, Cu_8/TiO_2, Ru_8/TiO_2, Rh_8/TiO_2, Pd_8/TiO_2, Ag_7/TiO_2, Os_8/TiO_2, Ir_8/TiO_2, Pt_8/TiO_2 and Au_7/TiO_2, respectively.

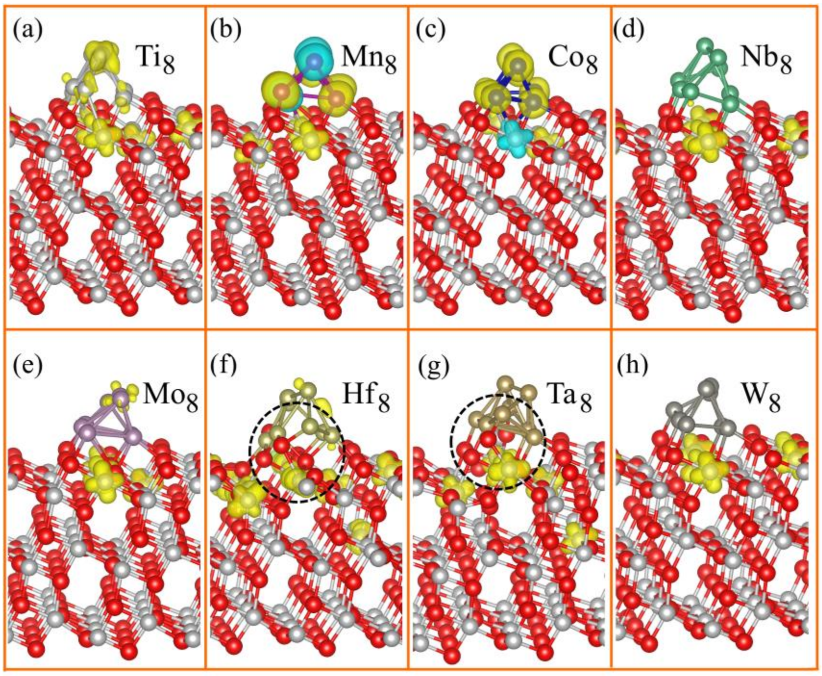

Supplementary Fig. 2. Optimized structures and spin density plots (at the iso-value of 0.015 $|e|/Bohr^3$) for active metals (electron affinity <1 eV) supported on TiO_2. In all cases, significant electron donation from metal to TiO_2, forming many Ti^{3+} cations, is observed upon interface formation. Particularly, the deposition of Hf and Ta clusters on TiO_2 causes intensive structural distortion at the interface, as one can see the off-lattice oxygens indicated by dash circles in panels (f) and (g).

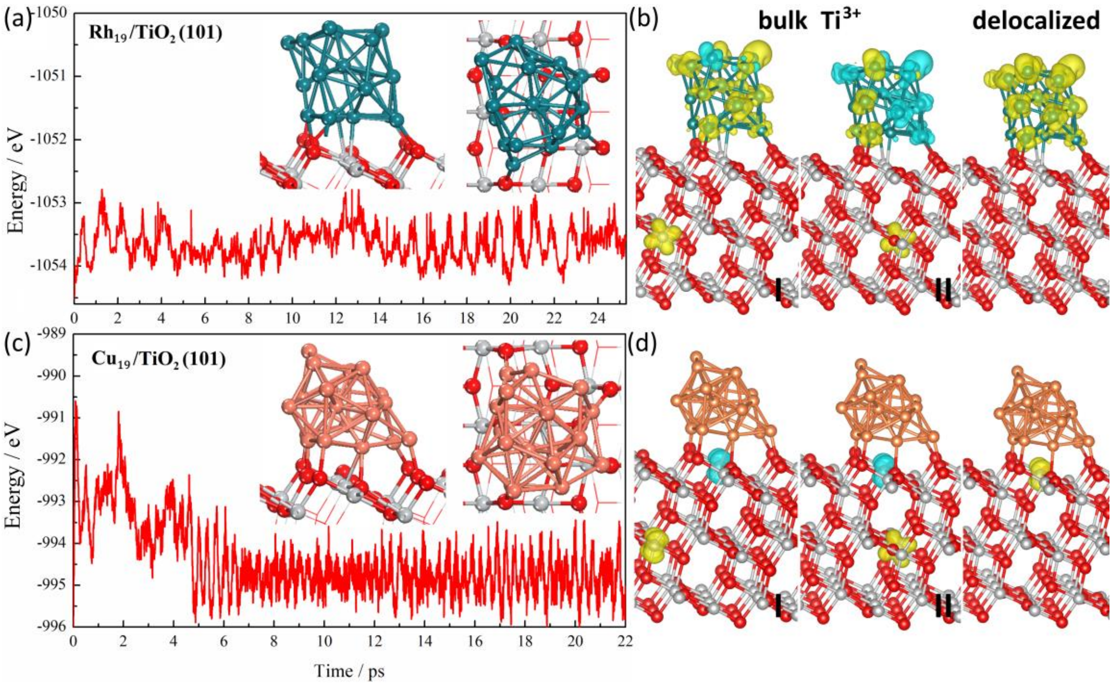

Supplementary Fig. 3. AIMD simulation trajectories (> 20 ps) as well as the side and top views of obtained optimal structures for (a) $Rh_{19}/TiO_2(101)$ and (c) $Cu_{19}/TiO_2(101)$ composites. Spin density plots (at the iso-value of 0.005 |e|/$Bohr^3$) evidencing the electron transfer from TiO_2 bulk (sites I and II) to metals for (b) $Rh_{19}/TiO_2(101)$ and (d) $Cu_{19}/TiO_2(101)$ composites are also presented.

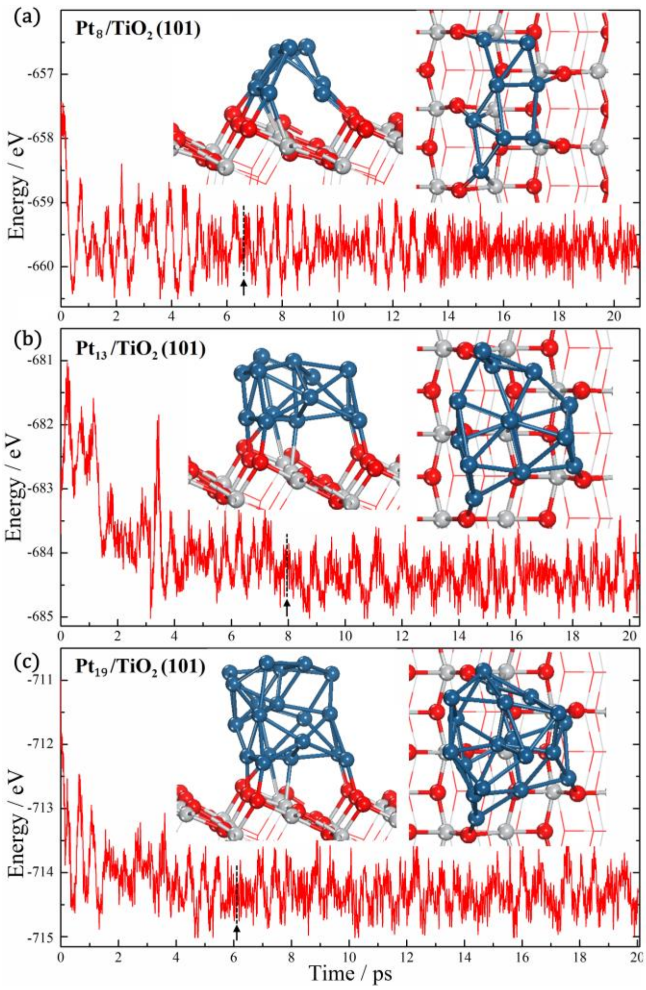

Supplementary Fig. 4. AIMD simulation trajectories as well as the obtained optimal structures for (a) $Pt_8/TiO_2(101)$, (b) $Pt_{13}/TiO_2(101)$ and (c) $Pt_{19}/TiO_2(101)$ composites. The structure equilibration generally occurs within ~8 ps in AIMD simulation as indicated by black arrows. Both the side and top views of the optimal Pt/TiO_2 structures are illustrated. Adapted with permission from ref. 28 (Wang, D., Liu, Z. P. & Yang, W. M. Revealing the Size Effect of Platinum Cocatalyst for Photocatalytic Hydrogen Evolution on TiO_2 Support: A DFT Study. *ACS Catal.* 8, 7270-7278 (2018)). Copyright (2018) American Chemical Society.

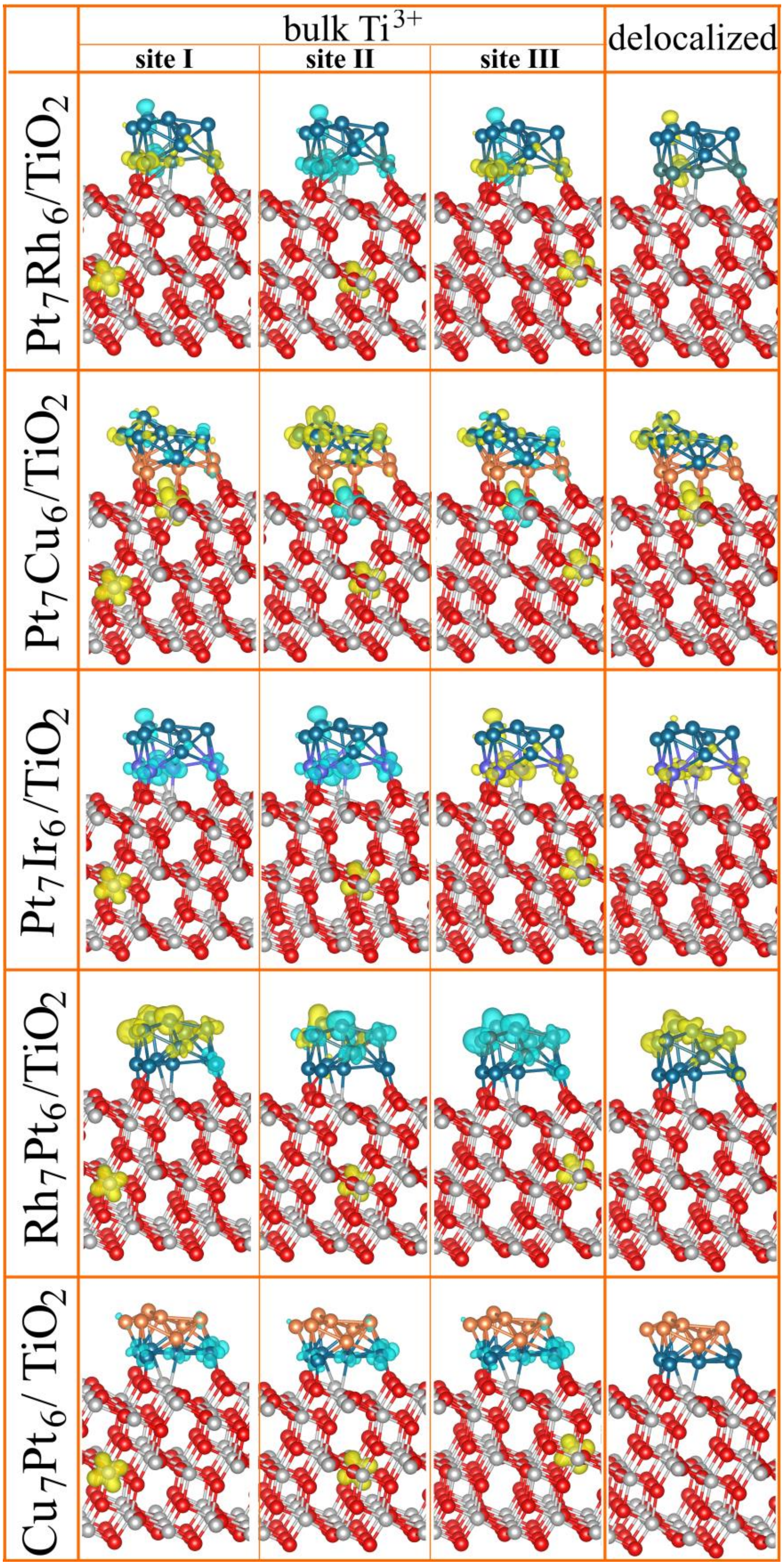

Supplementary Fig. 5. Spin density plots (at the iso-value of 0.005 |e|/Bohr^3) evidencing the electron transfer from TiO_2 bulk to metals for (a) Pt_7Rh_6/TiO_2, (b) Pt_7Cu_6/TiO_2, (c) Pt_7Ir_6/TiO_2, (d) Rh_7Pt_6/TiO_2 and (e) Cu_7Pt_6/TiO_2 composites, respectively. Bader charge analysis also confirmed

an increasing electron quantity of ~0.4 |e| on metals for the delocalized state relative to the initial bulk trapping state. Considering many possible electron trapping sites in the subsurface region of TiO_2, we have calculated three different electron trapping sites (Sites I, II, and III) for each composite and the reported IET energies were the averaged values.

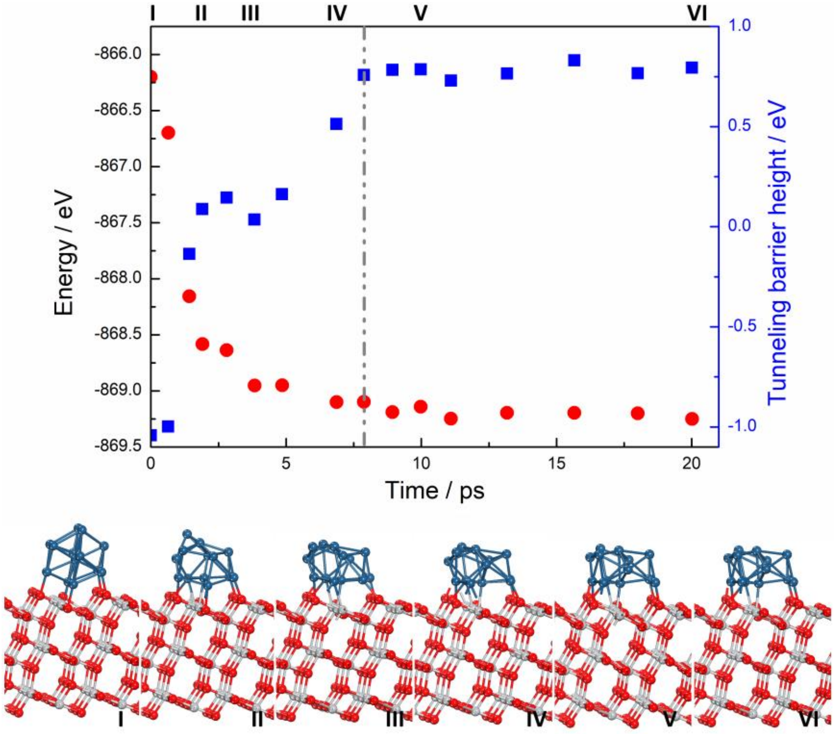

Supplementary Fig. 6. Calculated system energies (red dots) and tunneling barrier heights (via HSE06 functional; blue squares) of several optimized snapshots in the AIMD trajectory of the Pt_{13}/TiO_2 composite. The geometries of six representative samples are presented in chronological order (from I to VI), showing the structural evolution of the Pt_{13}/TiO_2 composite. The Pt_{13} initial structure (image I) was obtained by depositing an icosahedral Pt_{13} (the most stable structure in the gas-phase) onto the $TiO_2(101)$ surface, which gradually evolves into a low-symmetry two-Pt-layer architecture (images II to VI). The system energy (red dots) decreases while Φ_{TB} (blue squares) increases sharply within the first ~3 ps period of AIMD simulations, and the variation tendency slows down in the 3~8 ps interval and roughly reaches convergence after ~8 ps. In addition, we observed significant difference of ~3 eV in thermostability and ~1.7 eV in Φ_{TB} between the initial and equilibrated structures. The results explicitly signify the importance of determining stable metal/oxide interfaces on reliable calculation results.

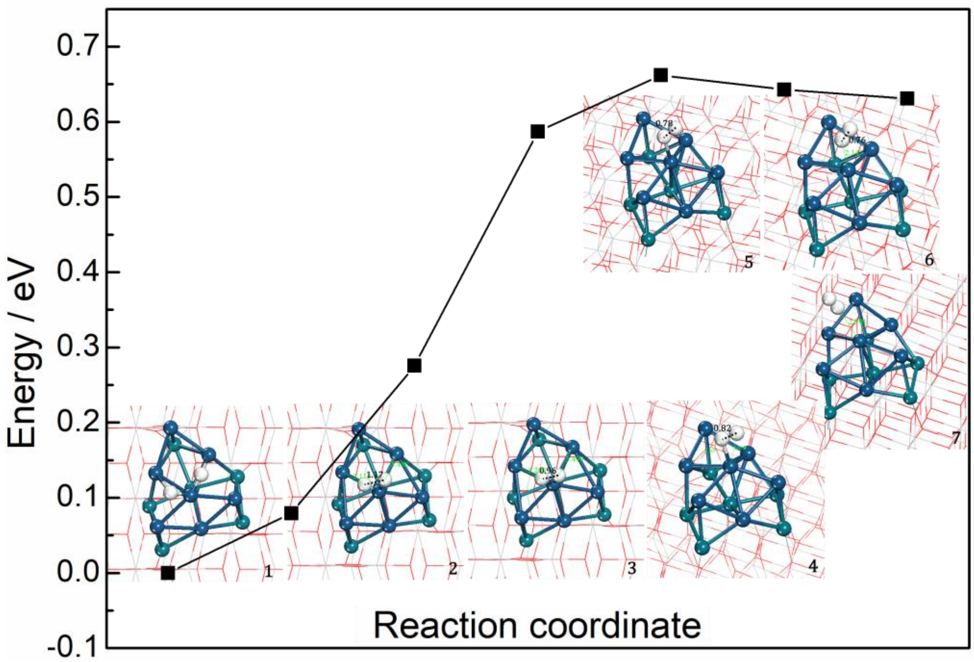

Supplementary Fig. 7. Geometry structures and energy profile for the H-H coupling process on Pt_7Rh_6/TiO_2 composites using the CI-NEB method. A very late transition structure with an energy barrier of 0.67 eV and transition distance of 0.78 Å (image 5), resembling the H_2 adsorption configuration, was determined.

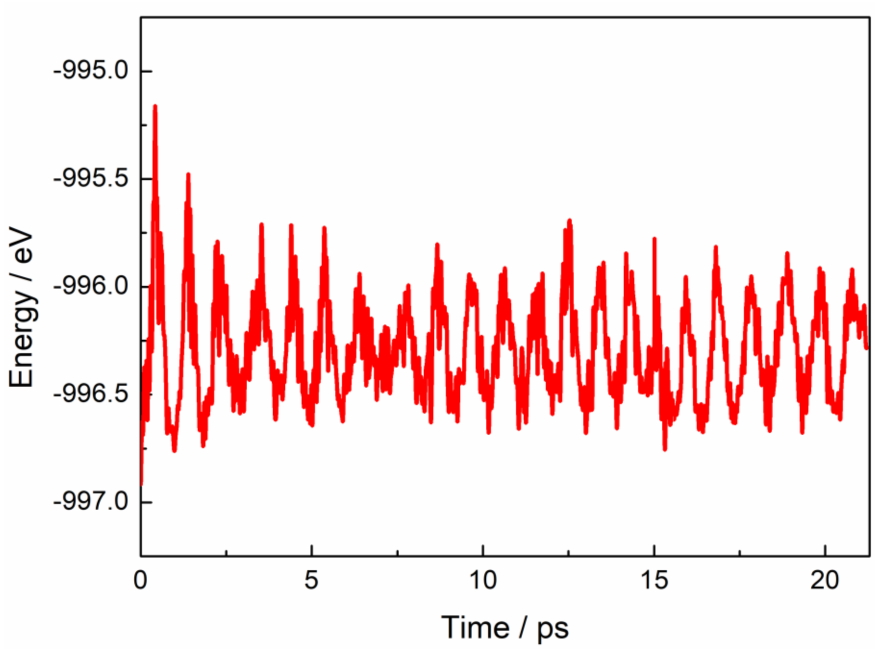

Supplementary Fig. 8. AIMD simulation trajectory for the $Pt_7Cu_6/TiO_2(101)$ composite under experimental hydrothermal treatment temperature of 450 K. We have selected 9 structural snapshots from the equilibrated trajectory (after 2.5 ps) in every 2 ps interval and fully optimized them until all forces diminish. From these optimized structures, no obvious changes in geometries or energies (energy difference less than 0.1 eV) were observed, evidencing the good stability of the Pt_7Cu_6/TiO_2 composite.

2. Supplementary Tables

Supplementary Table 1. Computed IET energies, tunneling barrier height Φ_{TB} (via hybrid HSE06 functional), and metal-TiO_2 interface distance (the vertical distance between metals and TiO_2) of considered metal/TiO_2 composites.

Composites	Φ_{TB} / eV	IET energy / eV	Distance / Å	Composites	Φ_{TB} / eV	IET energy / eV	Distance / Å
Pt_5/TiO_2	-2.18	-0.39	1.11	Cu_7Pt_6/TiO_2	0.76	0.02	1.56
Pt_8/TiO_2	-0.72	-0.17	1.24	Cu_{19}/TiO_2	0.79	0.01	1.51
Pt_{13}/TiO_2	0.83	0.05	1.56	Pt_6Cu_{13}/TiO_2	0.70	-0.05	1.57
Pt_{19}/TiO_2	0.90	0.01	1.62	Ir_8/TiO_2	-1.05	-0.08	1.28
Rh_5/TiO_2	-2.80	-0.39	1.10	Pt_7Ir_6/TiO_2	0.01	0.09	1.52
Rh_8/TiO_2	-1.40	-0.27	1.19	Os_8/TiO_2	-2.06	-0.23	1.20
Rh_{13}/TiO_2	0.80	-0.08	1.48	Pd_8/TiO_2	-0.10	-0.08	1.24
Pt_7Rh_6/TiO_2	0.54	-0.04	1.54	Pd_{13}/TiO_2	1.70	0.09	1.60
Rh_7Pt_6/TiO_2	1.00	0.07	1.56	Ag_7/TiO_2	3.00	0.23	1.96
Rh_{19}/TiO_2	0.40	-0.05	1.62	Au_7/TiO_2	2.79	0.21	1.89
Cu_8/TiO_2	-0.46	-0.15	1.18	Ni_8/TiO_2	/	0.09	1.02
Cu_{13}/TiO_2	0.75	-0.08	1.52	Ru_8/TiO_2	/	0.01	1.10
Pt_7Cu_6/TiO_2	0.60	-0.08	1.44				

Supplementary Table 2. Gibbs adsorption energies of H atom (ΔG_H) at various metal sites on Pt_7Rh_6/TiO_2, Pt_7Cu_6/TiO_2 and Pt_6Cu_{13}/TiO_2 composites, respectively. The H adsorption favors the bridge configuration involving two Pt atoms. Two of the most reactive sites with exothermic ΔG_H approaching zero on each composite are indicated in bold. Energy unit: eV.

Items / Sites	Pt_7Rh_6/TiO_2	Pt_7Cu_6/TiO_2	Pt_6Cu_{13}/TiO_2
a	**-0.02**	**-0.01**	**-0.07**
b	**-0.06**	**0.00**	**-0.10**
c	-0.12	-0.50	-0.54
d	0.01	-0.41	-0.47
e	-0.18	0.28	-0.14
f	-0.09	0.03	-0.28
g	-0.18	-0.34	-0.27
h	-0.11	0.25	-0.18
i	0.02	-0.57	\
j	-0.08	-0.50	\
